# Lifestyle modification in atrial fibrillation: Mechanisms, phenotypes and ablation outcomes

**DOI:** 10.1113/EP093386

**Published:** 2026-02-23

**Authors:** Konstantinos Grigoriou, Paschalis Karakasis, Panagiotis Theofilis, Konstantinos Pamporis, Panayotis K. Vlachakis, Anastasios Apostolos, Nikolaos Ktenopoulos, Barbara Fyntanidou, Efstratios Karagiannidis, Dimitrios Patoulias, Antonios P Antoniadis, Nikolaos Fragakis

**Affiliations:** ^1^ Department of Pharmacology, School of Medicine National and Kapodistrian University of Athens Athens Greece; ^2^ Second Department of Cardiology, Hippokration General Hospital Aristotle University of Thessaloniki Thessaloniki Greece; ^3^ First Cardiology Department, School of Medicine, Hippokration General Hospital National and Kapodistrian University of Athens Athens Greece; ^4^ Department of Cardiology Guy's and St Thomas’ NHS Foundation Trust, Harefield Hospital London UK; ^5^ Emergency Department, AHEPA University General Hospital Aristotle University of Thessaloniki Thessaloniki Greece; ^6^ Second Propedeutic Department of Internal Medicine, Faculty of Medicine, School of Health Sciences Aristotle University of Thessaloniki Thessaloniki Greece

**Keywords:** atrial fibrillation, catheter ablation, lifestyle modification, obesity, physical activity, sleep apnoea

## Abstract

Atrial fibrillation (AF) is the most prevalent sustained cardiac arrhythmia and is associated with significant morbidity, mortality and healthcare utilization. Catheter ablation is increasingly used as a rhythm‐control intervention for patients with symptomatic paroxysmal and persistent AF, yet recurrence rates remain suboptimal. This finding can be partly explained due to the rising prevalence of AF risk factors, such as obesity, sedentary lifestyle, sleep apnoea, diabetes, hypertension and other modifiable lifestyle‐related contributors. Many of these drivers are potentially reversible, and growing evidence indicates that addressing them can improve post‐ablation outcomes. The incorporation of lifestyle and risk factor management into a structured, protocol‐driven, multidisciplinary AF care programme may maximize these benefits. This review underscores the interplay between modifiable lifestyle risk factors and post‐ablation outcomes, explores the underlying mechanistic pathways and phenotypes, and evaluates the impact of lifestyle interventions. In addition, it provides practical guidance on peri‐ablation strategies and discusses the role of imaging and digital tools. Key implications for clinical practice, existing knowledge gaps and directions for future research are also discussed.

## Abstract (highlights)

- **What is the topic of this review?**
  How modifiable lifestyle factors shape atrial fibrillation (AF) pathobiology, phenotypes and clinical management, including effects on ablation outcomes, and the role of imaging and digital tools.
- **What advances does it highlight?**
  An integrative framework linking epicardial adipose tissue, inflammation, and autonomic/haemodynamic remodelling; evidence for multidisciplinary, pre/post‐ablation risk‐factor programmes; and actionable targets – weight loss, fitness, alcohol moderation, obstructive sleep apnoea screening/continuous positive airway pressure – with phenotype‐tailored, digitally enabled follow‐up to reduce AF burden, re‐ablation and antiarrhythmic dependence.

## 1 INTRODUCTION

Atrial fibrillation (AF) not only remains the most common cardiac arrhythmia worldwide, but its prevalence and incidence are projected to rise because of the population ageing, more efficient treatment of chronic diseases, advancements in diagnostic tools and increasing incidence of associated comorbidities (Kornej et al., 2020; Tanaka et al., 2021). Globally, the estimated number of people suffering from AF is 59 million, with a lifetime risk of 1 in 3–5 individuals aged above 45 (Kornej et al., 2020). AF places a growing burden on healthcare resources, as it is associated with an increased risk of thromboembolic events, heart failure (HF) and mortality (Patel et al., 2014). In parallel with oral antithrombotic therapy, the restoration and preservation of sinus rhythm are considered fundamental components of AF management (Kirchhof et al., 2020). Catheter ablation of AF is currently positioned as a common and effective intervention for the treatment of paroxysmal and persistent AF (Van Gelder et al., 2024). However, AF recurrences are common, affecting 20–50% of patients, and approximately 1 in 8 individuals are expected to undergo repeat AF ablation within a year (Al‐Hijji et al., 2016; Calkins et al., 2017). Therefore, in order to improve the long‐term success of this procedure, treatment approaches tailored to individual characteristics and AF‐related comorbidities are of significant importance.

**FIGURE 1:**
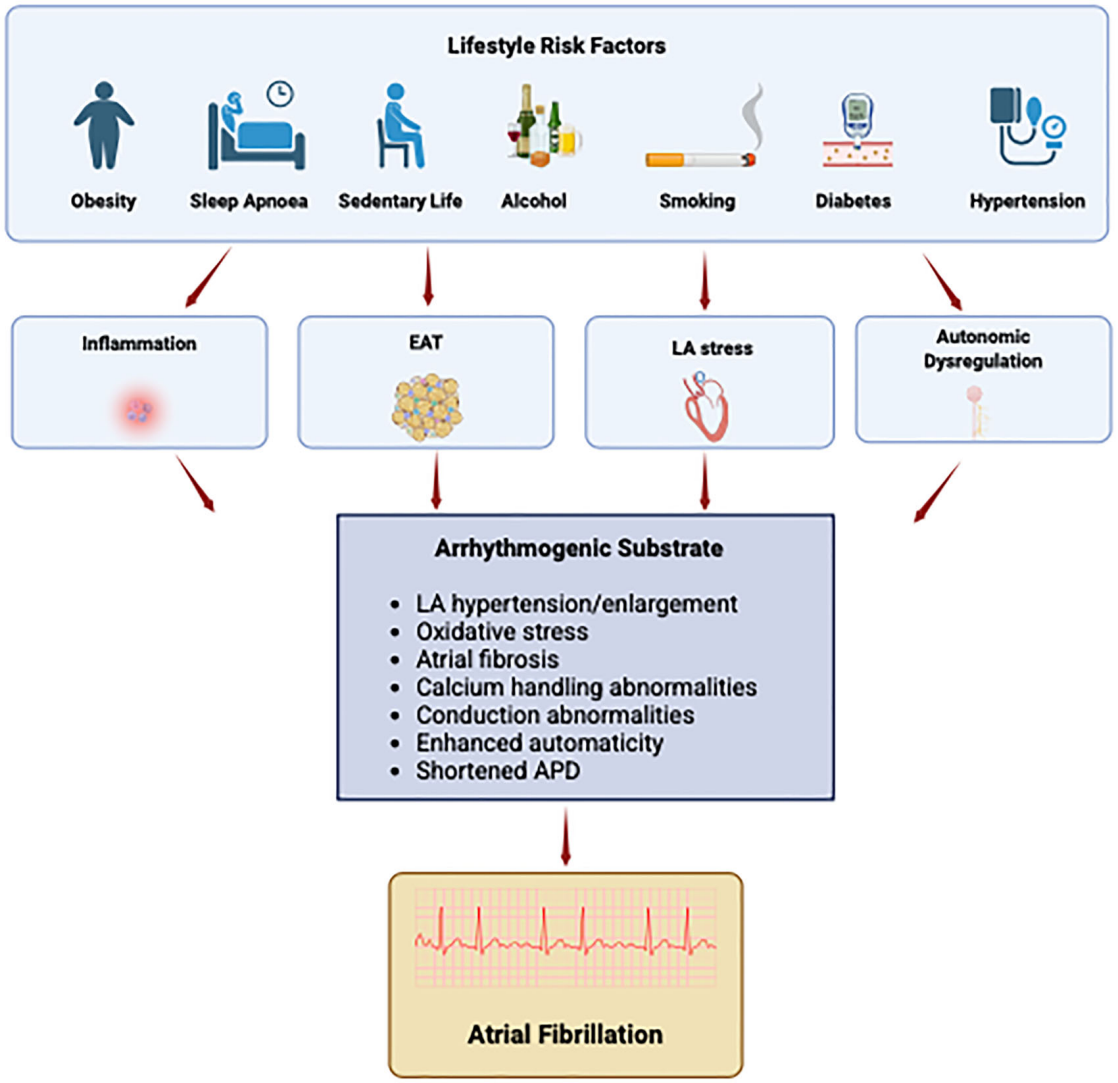
Pathophysiological drivers of lifestyle risk factors in atrial fibrillation. Lifestyle risk factors, including obesity, sleep apnoea, sedentary behaviour, alcohol, smoking, diabetes and hypertension, promote AF through multiple upstream mechanisms such as inflammation, EAT accumulation, LA stress and autonomic dysregulation. These processes create an arrhythmogenic substrate characterized by LA enlargement, oxidative stress, atrial fibrosis, conduction abnormalities, enhanced automaticity and shortened APD, ultimately promoting both the initiation and maintenance of AF. APD, action potential duration; EAT, epicardial adipose tissue; LA, left atrial.

The onset and progression of AF are significantly related to numerous modifiable lifestyle risk factors, such as diabetes mellitus (DM), dyslipidaemia, obesity, hypertension, obstructive sleep apnoea (OSA), excessive alcohol use, sedentary behaviour and tobacco use (Vermeer et al., 2024) (Figure 1). These determinants also contribute to and encompass the cardiovascular–kidney–metabolic (CKM) syndrome, a relatively new clinical entity that reflects the multifaceted interactions among the heart, kidneys and metabolic disorders (Ndumele et al., 2023). AF commonly arises as a prevalent sequela of CKM syndrome, thus placing it within the spectrum of cardiometabolic diseases. Addressing most of these factors has been shown to result in significant reductions in AF symptoms, improved maintenance of sinus rhythm and reverse atrial cardiomyopathy (Abed et al., 2013; Donnellan et al., 2020; Larstorp et al., 2019; Nalliah et al., 2022; Pathak et al., 2014, 2015; Teraoka et al., 2024; Voskoboinik et al., 2020). Nevertheless, the results of these trials should be interpreted and generalized to the AF population with caution due to their methodological limitations, primarily stemming from their non‐randomized design.

The role of lifestyle therapy as an AF‐modifying strategy, particularly in the context of catheter ablation, has been a subject of ongoing investigation. An increasing body of evidence seeks to clarify the optimal timing of lifestyle modification in relation to catheter ablation, as well as whether these changes alone or in combination with antiarrhythmic drugs (AADs), may be sufficient. The PRAGUE‐25 trial demonstrated that catheter ablation was superior to lifestyle changes combined with AADs in maintaining sinus rhythm, while the SORT‐AF trial did not decrease AF burden for patients who lost weight after catheter ablation for AF (Gessler et al., 2021; Osmancik et al., 2025). In contrast, a recent study showed that integrated lifestyle modification before AF ablation reduced both direct cardioversions and repeated ablations by approximately 50% in a period of 12 months after the procedure (Vermeer et al., 2025). The mixed evidence highlights the need for additional investigation (Table 1).

**TABLE 1:** Key studies showing evidence for lifestyle risk factor management in patients undergoing AF catheter ablation.

Study	Study type	Publication year	Number of patients	Population	Intervention	Comparator	Follow‐up (Months)	Outcomes
ARREST‐AF (Pathak et al., 2014)	Cohort	2014	149	Patients with AF, BMI > 27 kg/m^2^ and ≥1 cardiac risk factor	Aggressive management of obesity, hypertension, OSA, smoking, alcohol, DM, after AF ablation	Usual care	41.9	Decreased AF symptom burden (*P *< 0.001) Improved arrhythmia‐free survival: 87% arrhythmia free treatment group vs. 17% in control group (*P *< 0.001)
SORT‐AF (Gessler et al., 2021)	Randomized, multicentre, controlled trial	2021	133	Patients with AF and BMI 30–40 kg/m^2^	Weight reduction post‐ablation	Usual care	12	AF burden reduced in both groups post‐ablation (*P *< 0.001) without difference between groups
POP‐AF (Vermeer et al., 2025)	Prospective, randomized, controlled	2025	145	Patients with AF and ≥1 modifiable risk factor (BMI > 27 kg/m^2^, hypertension, DM, dyslipidaemia, smoking, alcohol)	Nurse‐led lifestyle treatment	Standard pre‐ablation counselling	12	Reduction of both repeat ablations (*P* = 0.045) and direct current cardioversions (*P* = 0.031)
PRAGUE‐25 (Osmancik et al., 2025)	Randomized, multicentre, noninferiority	2025	212	Patients with AF and BMI 30–40 kg/m^2^	Lifestyle modification (weight loss, increase in physical activity, alcohol reduction) in combination with AADs	Catheter ablation	23.5	Ablation (73.0% AF‐freedom at 12 months) superior to lifestyle modification combined with AADs (34.6% AF‐freedom at 12 months)

Abbreviations: AAD, antiarrhythmic drug; AF, atrial fibrillation; BMI, body mass index; DM, diabetes mellitus.

The aim of this review is to reinforce our knowledge and awareness of the accumulating data related to the management of AF through health behaviour changes and risk factor modification. We briefly discuss the underlying pathophysiological pathways that identify AF as a cardiometabolic disorder and focus on the potential effects of lifestyle interventions on improving AF ablation outcomes. Finally, clinical recommendations are provided to be incorporated into a holistic and personalized approach, as advocated by modern scientific practice.

## 2 PATHOBIOLOGY: CONVERGENT CARDIOMETABOLIC MECHANISMS

### 2.1 Atrial adiposity and ectopic fat

Excessive and dysfunctional adiposity acts as the primary trigger of the pathophysiological cascade underlying cardiometabolic disorders, which is characterized by inflammation and metabolic imbalance, both of which promote arrhythmogenesis (Bode et al., 2024; Boonstra & Post, 2004; Dai & Rabinovitch, 2009; Guo et al., 2012). Pericardial adipose tissue consists of two distinct types of visceral white adipose tissue, epicardial adipose tissue (EAT) and paracardial adipose tissue, each with different embryological origins (Gaborit et al., 2017). Most of the evidence linking cardiac fat to AF comes from studies focused on EAT (Gawałko et al., 2023). The accumulation and phenotypic changes of peri‐atrial EAT have been identified as key regulators of AF pathophysiology (Karakasis et al., 2025). EAT is a highly secretory and active fat with rather cardioprotective properties in healthy individuals (Iacobellis, 2015). It releases bioactive products such as free fatty acids, peptides, adipocytokines, growth factors and inflammatory signalling molecules, which regulate fibrotic, inflammatory, oxidative and electrophysiological processes within atrial tissue (Gawałko et al., 2023; Karakasis et al., 2025).

In pathological conditions such as obesity, DM, HF and AF, the secretome of EAT undergoes a shift toward a proinflammatory and profibrotic phenotype, exerting adverse effects on the heart (Karakasis et al., 2025). At this stage, peri‐atrial thickened and dysfunctional EAT releases bioactive modulators, such as matrix metalloproteinases (MMPs), angiopoietins and activin A, which promote fibrosis to spread from the epicardial layer to the subepicardial myocardium, resulting in impaired atrial structural remodelling (Gawałko et al., 2023; Karakasis et al., 2025). EAT also secretes a wide range of chemokines, including interleukin (IL)‐6, IL‐8, IL‐1 and monocyte chemoattractant protein‐1 (MCP‐1), which induce local inflammation and contribute to the formation of an arrhythmogenic substrate (Gawałko et al., 2023; Karakasis et al., 2025). Beyond these paracrine inflammatory effects, EAT‐derived extracellular vesicles enriched in arrhythmogenic microRNAs (miRNAs), particularly miR‐1‐3p and miR‐133a‐3p, have been shown to directly impair cardiomyocyte electrophysiology by inducing conduction slowing and facilitating re‐entrant activity (Ernault et al., 2023). As disease advances, fibrotic tissue gradually replaces EAT, leading to a fibro‐fatty infiltration of the subepicardial myocardium, a process commonly observed in HF, mitral valvulopathy and AF (Karakasis et al., 2025). This fibro‐fatty remodelling induces a vulnerable substrate, characterized by slow and heterogeneous conduction, gap junction remodelling, and the emergence of re‐entrant circuits and rotors (Karakasis et al., 2025; Nalliah et al., 2020) (Figure 1).

### 2.2 Inflammation and oxidative stress

Inflammation is a well‐established mechanism in the initiation and perpetuation of AF, and its significance becomes even more evident when considered within the framework of the rising prevalence of metabolic disorders (Grigoriou et al., 2025; Guo et al., 2012). Elevated levels of pro‐inflammatory mediators such as IL‐6, IL‐2, IL‐8, tumour necrosis factor‐α (TNF‐α), C‐reactive protein (CRP) and MCP‐1 have been consistently associated with the onset and recurrence of AF, even after rhythm control interventions (Guo et al., 2012; Koyama et al., 2009; Lim et al., 2014; Richter et al., 2012). Mechanistically, inflammation promotes alterations in ion channel expression and impairs gap junctions, both of which facilitate atrial electrical remodelling (Mukai, 2024). Simultaneously, circulating cytokines, including IL‐6 and TNF‐α, activate cardiac fibroblasts, resulting in enhanced fibrotic tissue formation, which contributes to the progression of atrial structural remodelling (Ihara & Sasano, 2022).

The atrial NOD‐like receptor protein 3 (NLRP3) inflammasome is a major contributor to AF onset, perpetuation and recurrence after catheter ablation (Dobrev et al., 2023; Han et al., 2022). It promotes the autocatalytic activation of caspase‐1 and the subsequent maturation and release of the effectors IL‐1β and IL‐18 (Han et al., 2022). A number of clinical conditions known to induce atrial cardiomyopathy, including DM, obesity and HF with preserved ejection fraction (HFpEF), converge mechanistically on NLRP3 inflammasome activation (Grigoriou et al., 2025; Karakasis et al., 2025). The inflammasome contributes to arrhythmogenesis through fibrosis, abnormal metabolism, gap junction remodelling, abnormal Ca^2+^ handling, ectopic beats and conduction defects (Poppenborg et al., 2025).

In addition to sterile inflammatory triggers, chronic systemic infections have become increasingly recognized as significant factors in the development of atrial cardiomyopathy (Karakasis et al., 2025). These conditions are associated with persistent low‐grade inflammation and endothelial dysfunction, which adversely modify atrial structure and pathophysiology, resulting in the formation of an arrhythmogenic substrate (Karakasis et al., 2025). For instance, HIV‐positive patients undergoing catheter ablation for AF had a notably higher burden of atrial scarring, more frequent triggers originating outside the pulmonary veins, and an increased rate of arrhythmia recurrence compared to matched HIV‐negative controls (La Fazia et al., 2023). Similarly, chronic *Helicobacter pylori* infection has been proposed as a potential contributor to atrial remodelling through systemic inflammation and immune‐mediated mechanisms, positioning it as a modifiable infectious risk factor in AF pathogenesis (Wang et al., 2025).

Oxidative stress, characterized by the overproduction of reactive oxygen species (ROS) and oxidative free radicals, represents a defining pathophysiological feature of metabolic disorders (Rani et al., 2016). ROS facilitate atrial arrhythmogenesis through various interrelated molecular mechanisms. For instance, these species oxidize ryanodine receptor‐2 (RyR2) and Ca^2+^‐calmodulin‐dependent protein kinase II (CaMKII), resulting in Ca^2+^ leakage in atrial myocardium (Grigoriou et al., 2025). ROS also enhance sarco/endoplasmic reticulum Ca^2+^‐ATPase (SERCA2) and protein kinase C (PKC) activation and impair nitroso‐redox balance, all of which further contribute to arrhythmogenesis (Grigoriou et al., 2025) (Figure 1).

### 2.3 Haemodynamic and structural remodelling of the left atrium

Left atrial (LA) hypertension and enlargement are common mechanistic endpoints of many cardiometabolic diseases that lead to AF (Al‐Kaisey & Kalman, 2021; Li et al., 2021). A consequence of these processes is increased atrial wall stress, which contributes to smooth muscle cell apoptosis and atrial fibrosis, driven predominantly by angiotensin II but also by transforming growth factor‐β (TGF‐β) and platelet‐derived growth factor (PDGF) (Markowitz, 2017; Schotten et al., 2011). Chronic stretch induces heterogeneous alterations in atrial structure, with electrophysiological consequences, which include prolongation of the effective refractory period and conduction slowing, thereby promoting enhanced AF inducibility (Hunter et al., 2012).

Structural remodelling of the atrium occurs in the setting of increased pressure and volume overload elicited by pathological stimuli such as hypertension and left ventricular (LV) diastolic dysfunction (Chen et al., 2021). Impaired LV stiffness and relaxation are present in HFpEF, predominantly driven by ageing, hypertrophy, and ischaemia (Gillebert et al., 2000). In a portion of patients, LV end‐diastolic pressure may be raised only during exercise (Borlaug et al., 2011). Finally, intrinsic atrial disorders, independent of haemodynamic variables, can decrease LA compliance and enhance LA stiffness, a condition known as stiff LA syndrome (Park et al., 2015) (Figure 1).

### 2.4 Autonomic dysregulation

Autonomic dysregulation plays a crucial role in AF pathophysiology. Both branches of the autonomic nervous system (ANS) can contribute to the initiation of AF (Mikhailov et al., 2025). Parasympathetic dominance shortens the action potential duration and effective refractory period and promotes re‐entry when triggers are present (Krapivinsky et al., 1995). Enhanced sympathetic nerve activity causes enhanced automaticity. Of note, the cardiac sympathetic nerves in AF are remodelled and designed, causing sympathetic hyperinnervation (Vandenberk et al., 2024). Cardiometabolic risk factors such as hypertension, DM, obesity, metabolic syndrome and OSA are associated with enhanced sympathetic activation (Esler, 2000; Narkiewicz et al., 1998; Nonogaki, 2000; Schlaich et al., 2015; Thorp & Schlaich, 2015). This leads to increased catecholamine levels, which affect Ca^2+^ homeostasis and its spontaneous release from the sarcoplasmic reticulum, which in turn promotes arrhythmogenesis via enhanced automaticity (Landstrom et al., 2017). In addition, the hyperactive sympathetic tone activates the renin–angiotensin–aldosterone system, leading to atrial structural remodelling and creating an arrhythmogenic substrate that facilitates atrial ectopies and focal triggers (Aguilar‐Gallardo et al., 2022). Furthermore, dietary habits, such as excessive alcohol consumption, and sleep deprivation or fragmentation may also predispose to AF episodes through ANS changes (Brunner et al., 2021; Ferreira et al., 2024) (Figure 1).

Heart rate variability (HRV) is one of the most widely applied methods to evaluate ANS fluctuations affecting the heart (Sassi et al., 2015). It is defined as the physiological variation in the time intervals between consecutive heartbeats and reflects the dynamic interplay between sympathetic and parasympathetic modulation of cardiac autonomic function (Goldberger, 1999). Elevated HRV indicates adaptability to internal and external stimuli, while reduced HRV represents a dysfunctional ANS with an impaired ability to maintain homeostasis (Boudet et al., 2017). HRV metrics have been shown to change markedly before the onset or recurrence of AF. In the ARIC cohort of 11,715 middle‐aged adults followed for a mean of 19.4 years, lower resting short‐term HRV indices derived from 2‐min ECG recordings were associated with an increased risk of incident AF, indicating that cardiac autonomic dysfunction precedes AF onset (Agarwal et al., 2017). Similarly, a meta‐analysis of 16 studies including 2352 patients demonstrated that HRV parameters predict AF recurrence after catheter ablation, highlighting the role of autonomic modulation in post‐ablation outcomes (Zhang et al., 2023). Hence, it may serve as a non‐invasive digital biomarker to predict AF burden and recurrence, both before and after AF episodes and related interventions (Zhang et al., 2025).

Another useful tool for assaying the function of the ANS, especially parasympathetic nerve function, is the evaluation of baroreflex sensitivity (BRS), which reflects the ability of the baroreflex to regulate heart rate in response to blood pressure (BP) changes (La Rovere et al., 2008). Impaired BRS has been consistently linked to increased AF burden. Transient elevations in BRS preceding AF onset have been reported, supporting the existence of a predominantly vagally mediated AF subtype (de Castro et al., 2006). Conversely, BRS is markedly reduced during AF episodes but appears to recover following rhythm control treatments (Field et al., 2016). Additionally, when assessed during sinus rhythm prior to catheter ablation, patients with persistent AF exhibit significantly lower BRS compared with those experiencing paroxysmal AF, which indicates a correlation between BRS and chronicity (Miyoshi et al., 2020).

Evaluation of HRV and BRS provides valuable information in patients with AF‐related lifestyle risk factors, especially for risk stratification. Low HRV and enhanced sympathetic activity have been described after full‐night sleep deprivation (Zhong et al., 2005). Sympathetic dominance has also been reported in acute sleep deprivation, as indicated by the low‐frequency/high‐frequency ratio of HRV, impaired adaptive responses to orthostatic challenges and reduced parasympathetic regulation (Al‐Hijji et al., 2016). Furthermore, enhanced low‐frequency components of BP variability (BPV) and reduced BRS have been reported after sleep deprivation (Zhong et al., 2005). Regarding alcohol consumption, acute alcohol ingestion has been associated with decreased short‐term HRV and an enhanced low‐frequency/high‐frequency ratio of HRV (Koskinen et al., 1994; Ralevski et al., 2019; Süfke et al., 2009). Moreover, high‐frequency HRV, indicative of increased parasympathetic nervous system activity, has been reported in moderate drinkers (Quintana et al., 2013). Of note, acute alcohol withdrawal was associated with a severe downregulation of BRS (Bär et al., 2006). These abnormalities in HRV and BRS likely represent intermediate markers linking lifestyle‐related autonomic imbalance to increased AF susceptibility, supporting their role in risk stratification rather than direct causal inference.

## 3 PHENOTYPING TO TARGET LIFESTYLE THERAPY

### 3.1 Clinical and biomarker phenotypes

Obesity is a well‐established risk factor for AF, and with its prevalence steadily rising worldwide, the number of obesity‐related AF cases is expected to rise accordingly (Zia et al., 2021). Both anthropometric indicators of general obesity (such as body mass index [BMI], body weight, and body fat percentage) and measures of abdominal obesity (such as waist circumference, waist‐to‐hip ratio, and waist‐to‐height ratio) have been demonstrated to be positively associated with the risk of AF (Aune et al., 2017; Fenger‐Grøn et al., 2017; Frost et al., 2014; Karas et al., 2016; Tchernof & Despres, 2013; W. Wang et al., 2004). Interestingly, with respect to fat distribution, while abdominal fat is typically associated with worse outcomes in most cardiometabolic diseases, this association is less evident in AF, where general obesity appears to represent a more significant AF risk factor than abdominal adiposity (Zia et al., 2021).

Beyond their impact on AF incidence, anthropometric measures, such as height, weight, BMI, body surface area and lean body mass, are significant predictors of clinical outcomes in patients with established AF (Boriani et al., 2022). Lower values of these parameters, corresponding to the lowest tertiles, are associated with increased all‐cause mortality, consistent with the obesity paradox observed in many cardiovascular (CV) diseases. Patients with lower body weight are more likely to represent a frail population with a higher burden of comorbidities, which contributes to poorer outcomes (Boriani et al., 2022).

Height is a nonmodifiable anthropometric factor that influences AF outcomes. In patients with drug‐refractory paroxysmal AF undergoing first‐time catheter ablation, greater height was associated with a higher risk of AF recurrence, with this relationship being particularly pronounced in female patients. Long‐term follow‐up demonstrated that increased height independently predicted post‐ablation AF recurrence in women. Imaging findings further suggested a link between body height and LA dimensions in females, indicating that height‐related atrial remodelling may contribute to a more arrhythmogenic substrate and reduced ablation durability (Liu et al., 2022).

Among the biomarkers implicated in AF, CRP and N‐terminal pro‐B‐type natriuretic peptide (NT‐proBNP) are established indicators reflecting inflammatory and haemodynamic processes, both of which are closely linked to cardiometabolic diseases. CRP levels are affected by multiple factors, including lifestyle habits, ageing, and metabolic disorders such as obesity and DM (Aronson et al., 2004; Liu et al., 2019; Thorand et al., 2003). The available evidence to date indicates that increased levels of CRP function as a prognostic factor in AF rather than as a causal contributor (Aviles et al., 2003; Li et al., 2022).

It has been widely acknowledged that NT‐proBNP is also elevated in patients with AF (Goetze et al., 2006). As HF commonly coexists with AF, it remains uncertain the degree to which the association between NT‐proBNP and outcomes reflects AF itself or the underlying burden of HF (Nasab Mehrabi et al., 2023). Nevertheless, a relatively recent study indicates that increased NT‐proBNP levels can predict future HF events in AF patients regardless of the presence of HF. Therefore, its measurement is recommended as part of the routine assessment of AF patients (Brady et al., 2022).

Furthermore, dyslipidaemia, typically characterized by elevated plasma total cholesterol, low‐density lipoprotein cholesterol (LDL‐c), triglycerides and reduced high‐density lipoprotein cholesterol (HDL‐c), induces systemic inflammation, oxidative stress, and autonomic dysfunction, all of which contribute to AF pathogenesis (Mauriello et al., 2025). However, although preclinical studies suggest that serum lipids contribute to AF onset, population‐based investigations have paradoxically reported an inverse relationship between lipid levels and AF incidence (Mauriello et al., 2025). Of note, lipoprotein(a) appears to be the only lipid biomarker with robust causal evidence for the risk of AF (Jiang et al., 2021; Singh et al., 2024).

### 3.2 Imaging phenotypes

Advanced imaging modalities have enabled detailed characterization of structural and tissue‐based phenotypes in AF, including LA volume and fibrosis, as well as quantification of epicardial and periatrial fat using computed tomography (CT) scans and cardiovascular magnetic resonance (CMR).

LA strain has emerged as a robust emerging imaging marker in AF, with the ability to capture subtle abnormalities beyond conventional measures of atrial size and volume (Donal et al., 2017). Abnormal LA strain parameters are considered valuable predictors of AF (Hauser et al., 2021). Reduced reservoir and conduit strain have been demonstrated in patients with type 2 DM (T2DM), even in the absence of overt structural heart disease, suggesting early atrial involvement (Benchea et al., 2025). Consistent with this, abnormal indices of diastolic function have also been observed in adolescents and young adults with obesity and T2DM, where LA strain analysis demonstrated reductions in reservoir, conduit and booster strain despite normal LA volume (Steele et al., 2020). Moreover, following catheter ablation for long‐standing AF, recovery of LA function is greater in patients who maintain sinus rhythm, with contractile strain at 3 months proving to be a strong determinant of recurrence (Khan et al., 2023). Taken together, these findings highlight the potential of LA strain to improve risk stratification, enable earlier detection of subclinical atrial myocardial disease, and guide therapeutic strategies in high‐risk populations.

CMR and CT have further advanced the phenotyping of AF by enabling precise assessment of EAT and periatrial adiposity. CMR provides highly accurate volumetric measurements of EAT and represents the gold standard for quantification, although its routine clinical use is limited by cost and time constraints (Flüchter et al., 2007; Gorter et al., 2008). A recently published study, which enrolled participants with CMR scans on record from the LOOP study, showed that atrial EAT was strongly linked to the incidence of subclinical AF (Guldberg et al., 2024). CT offers high‐resolution measurements of thickness, volume and total area, with three‐dimensional reconstruction enabling detailed characterization of adipose tissue (Tsao et al., 2011). CT‐based evaluations have highlighted a distinct relationship between EAT and AF, independent of general adiposity markers such as BMI and body surface area, as well as atrial size (Gaibazzi et al., 2021; Kusayama et al., 2016). Furthermore, increased pericoronary adipose tissue has been linked to AF recurrence following catheter ablation, likely reflecting underlying inflammatory processes (Nogami et al., 2021). Cardiac CT has also been used to characterize peri‐atrial fat. Patients with AF were shown to have greater peri‐atrial adipose tissue volumes, while higher fat burden and lower attenuation displayed only weak spatial connections with adverse electrophysiological remodelling (Vickneson et al., 2025). Together, quantitative assessment of EAT using these modalities has been associated with AF incidence and recurrence following ablation, underscoring its potential as an imaging biomarker that links metabolic risk with atrial remodelling.

Late gadolinium enhancement (LGE)‐CMR provides non‐invasive assessment of atrial fibrosis and offers prognostic insight for AF ablation outcomes (Karakasis et al., 2024). Although limited by resolution, variable protocols, and incomplete detection of diffuse endomysial fibrosis, a higher fibrosis burden on LGE‐CMR is strongly associated with increased arrhythmia recurrence and repeat ablation (Chelu et al., 2018; Karakasis et al., 2024). Fibrosis‐guided ablation using LGE‐CMR has been shown to reduce AF recurrence without increasing procedural complications, supporting the value of tissue‐targeted strategies in optimizing ablation (Salih et al., 2025).

### 3.3 Physiological and digital phenotypes

The integration of physiological and digital phenotyping into AF research has opened new avenues for more proper and personalized management of lifestyle factors that may influence AF onset, progression and ablation outcomes. Wearable device technologies, such as smartwatches and exercise bands, have become increasingly adopted by patients with AF, offering a non‐invasive means of continuously monitoring physical activity, oxygen saturation, heart rate and sleep quality (Hughes et al., 2023). These devices, through a single‐lead electrocardiogram (ECG), enable the measurement of HRV, which has predictive value for AF (Zhang et al., 2025; Zillner et al., 2023). In addition, they facilitate the collection of data relevant to the diagnosis of sleep disorders, which are negatively associated with AF (Deshmukh et al., 2025; Zheng et al., 2024).

Moreover, BPV is recognized as an independent predictor of future CV outcomes, including coronary artery disease, HF, stroke and all‐cause mortality, regardless of mean BP levels (Muntner et al., 2015; Poortvliet et al., 2012; Stevens et al., 2016; Suchy‐Dicey et al., 2013). Emerging evidence also suggests that BPV is independently associated with AF risk. Population‐based data show that greater systolic and diastolic BP variability is modestly linked to higher AF incidence after adjustment for traditional risk factors and average BP, irrespective of hypertension status or antihypertensive treatment (Lee et al., 2020). Similar associations have been reported in individuals with T2DM, underscoring the relevance of BP stability in high‐risk populations (Kaze et al., 2023). In parallel, increasing interest surrounds the use of automated BP devices for AF detection and monitoring, with potential implications for both clinical practice and research (Kollias et al., 2024).

Last, advances in continuous glucose monitoring (CGM) have improved the assessment of glycaemic control in DM. CGM tracks glucose levels in interstitial fluid over time, offering a more detailed view of daily fluctuations than haemoglobin A1C (HbA1C) (Nathan et al., 1993). One key metric derived from CGM, known as time in range (TIR), typically defined as glucose levels between 3.9 and 10.0 mmol/L, has been linked to microvascular complications and increased CV mortality (Battelino et al., 2019; Lu et al., 2018, 2021). Moreover, a relatively recent study demonstrated that TIR is independently associated with stroke risk in patients with AF and T2DM, a finding that highlights its potential role in improving CV risk stratification in this population (Guo et al., 2021).

### 3.4 Responder profiles and thresholds

The identification of responders and non‐responders to lifestyle interventions enables the early personalization of therapies, which will subsequently enhance the effectiveness of AF management. A central pillar of this approach is the implementation of adaptive interventions which meet the needs of each patient. Regarding weight management strategies, early non‐responders were distinguished from responders by lower positive mood, greater boredom, increased susceptibility to dietary temptations, and less alignment between their eating behaviours and weight management goals, despite comparable intervention adherence (Unick et al., 2019). In OSA, the evaluation of anatomical (upper‐airway collapsibility) and physiological (loop gain, airway muscle responsiveness and arousal response threshold) factors could help identify which patients are likely to benefit from continuous positive airway pressure (CPAP) therapy (Chu & Zinchuk, 2024). Another lifestyle‐related AF risk factor that needs to be addressed is alcohol. Assessment of patients’ motivation and confidence may assist in determining which patients would have a higher probability of responding positively to a moderation‐focused management approach (Kuerbis et al., 2014).

## 4 LIFESTYLE INTERVENTIONS: MECHANISTIC RATIONALE AND CLINICAL EVIDENCE

### 4.1 Weight loss

Weight loss through lifestyle changes or bariatric surgery can reverse obesity‐related cardiac remodelling and improve AF outcomes (Donnellan et al., 2019; Middeldorp et al., 2018). In overweight and obese individuals with a history of AF, losing at least 10% of body weight was associated with a sixfold greater chance of remaining free from arrhythmia compared with those who lost less. Patients whose weight dropped and then rebounded, resulting in more than a 5% change between annual assessments, had about double the likelihood of arrhythmia recurrence compared with those who maintained more consistent weight (Pathak et al., 2015).

The benefits of weight reduction were accompanied by favourable cardiac structural changes, such as smaller LA volumes, reduced ventricular hypertrophy, reduced inflammation and better glycaemic control, and were most effectively achieved in physician‐led programmes targeting weight and risk‐factor control (Pathak et al., 2015). Of note, in an animal model of obese sheep, a 30% weight loss was associated with reduced atrial EAT volume, atrial fat infiltration and reversal of fibrosis (Mahajan et al., 2021).

In clinical trials, aggressive weight management with bariatric surgery or risk factor modification has been shown to reduce the risk of AF development and AF recurrence after catheter ablation (Donnellan et al., 2019; Mahajan et al., 2021). Similarly, a multicentre randomized controlled trial (RCT) demonstrated that physician‐led lifestyle and risk factor management significantly improved freedom from AF and reduced recurrence of AF at 12 months after first‐time catheter ablation compared with usual care in patients with elevated BMI and cardiometabolic comorbidities (Pathak et al., 2025). Furthermore, a relatively recent meta‐analysis demonstrated that patients with ≥10% weight reduction, AF duration under 1 year and weight loss before the procedure had fewer AF recurrences (Park et al., 2022).

In addition to behavioural and surgical strategies, pharmacological approaches, such as glucagon‐like peptide 1 (GLP‐1) receptor agonists and dual GLP‐1/glucose‐dependent insulinotropic polypeptide (GIP) agonists, have shown substantial weight loss in clinical trials and represent an emerging adjunct to optimize AF outcomes in patients with obesity (Aronne et al., 2024; Karakasis et al., 2024, 2026; Rubino et al., 2021).

### 4.2 Obstructive sleep apnoea

OSA is a highly common and often overlooked sleep disorder with significant CV consequences, especially in patients with AF (Sousa et al., 2025). OSA involves recurrent episodes of upper airway obstruction during sleep, leading to apnoeas and hypopneas accompanied by drops in arterial oxygen saturation. This disorder is generally diagnosed when the apnoea–hypopnea index (AHI) is ≥5. Severity is classified as mild (AHI 5–14), moderate (15–29) and severe (≥30) (Berry et al., 2007). Cardiometabolic disorders such as obesity, hypertension, DM and metabolic syndrome are well‐established risk factors for OSA (Sankaranarayanan et al., 2023; Zhou et al., 2020).

A strong bidirectional relationship between OSA and AF has been shown in multiple studies. Reported OSA prevalence ranges widely, from 21% to 82% (Asirvatham & Kapa, 2009; Desteghe et al., 2018; Drager et al., 2018; Mehra et al., 2006; Todd et al., 2010). Despite variability in study designs, moderate‐to‐severe disease consistently emerged as a common pattern in patients with AF (Sousa et al., 2025). OSA worsens AF management outcomes by reducing the effectiveness of AADs and increasing recurrence after electrical cardioversion or catheter ablation (Huang et al., 2021). It is also linked to higher rates of postoperative AF after coronary artery bypass grafting (van Oosten et al., 2014).

Timely and accurate diagnosis of OSA in those with, or at risk for, AF is therefore crucial. Diagnostic strategies have ranged from in‐laboratory polysomnography (PSG) and home sleep apnoea tests to pacemaker‐based monitoring and screening tools like the STOP‐bang, Epworth sleepiness scale or monitoring, objective outcomes, and definitions in symptomatic atrial fibrillation (MOODS‐AF) (Kadhim et al., 2025; Sousa et al., 2025). However, there is no standardized, AF‐specific screening pathway, and routine incorporation of OSA assessment into AF care remains inconsistent. Indeed, underdiagnosis of OSA in patients with AF continues to be a major challenge, primarily resulting from limited awareness, the frequent absence of hallmark symptoms such as excessive daytime sleepiness among CV patients, and the limited effectiveness of existing diagnostic methods (Sousa et al., 2025).

CPAP therapy remains the gold standard for managing OSA and has been increasingly investigated for its impact on AF outcomes. Numerous observational studies have demonstrated that CPAP use reduces the incidence of AF recurrence compared with untreated patients (Li et al., 2014). However, randomized data are less conclusive. Evidence from a recent randomized controlled trial with a relatively small sample size indicated that CPAP therapy may not significantly influence arrhythmia recurrence within the first year after AF catheter ablation (Hunt et al., 2022). A similar finding was confirmed by another study of patients with severe OSA, where AF incidence during early follow‐up after ablation was not reduced in those using CPAP. Notably, in that same population, prolonged adherence to CPAP therapy was associated with a lower rate of AF recurrence over the long term (Tanaka et al., 2025). Mechanistic insights from the SLEEP‐AF study demonstrated that CPAP therapy in patients with OSA and AF can reverse atrial remodelling, improving conduction and reducing areas of complex fractionated electrograms (Nalliah et al., 2022). Collectively, these findings underscore the potential of CPAP adherence not only to modulate the atrial substrate but also to enhance personalized AF management, highlighting the need for strategies to improve long‐term patient adherence.

### 4.3 Alcohol reduction/abstinence

Alcohol consumption is linked to incident AF and adverse changes in atrial structure. Observational studies indicate a dose‐dependent relationship between alcohol consumption and AF risk, LA enlargement, LA fibrosis and AF recurrence after catheter ablation (Voskoboinik et al., 2016). A randomized study also showed that alcohol shortens pulmonary vein refractory periods (Marcus et al., 2021). Moreover, alcohol is directly associated with other risk factors for AF, including obesity, OSA, hypertension and HF (Lee et al., 2024).

Both acute and long‐term alcohol use, as well as past alcohol consumption, impact the onset and recurrence of AF in distinct ways (Wong et al., 2023). Acute alcohol consumption can trigger AF, with even a single drink doubling the odds of an episode and two or more drinks more than tripling the risk in individuals with paroxysmal AF (Marcus et al., 2021). Long‐term intake further increases AF risk in a dose‐dependent manner. Accumulating data indicate that each 84‐g weekly increase in alcohol raises AF risk by 8% and that moderate consumption (7–14 drinks per week) is also associated with higher AF risk, while lower levels appear less harmful (Gallagher et al., 2017; Larsson et al., 2014).

Accumulating evidence suggest that lowering alcohol intake can substantially reduce the incidence and recurrence of AF. Indeed, a RCT in regular alcohol consumers with paroxysmal or persistent AF demonstrated that reducing intake markedly improves outcomes. Participants assigned to abstinence decreased consumption from 17 to 2 drinks per week and experienced both a lower risk of AF recurrence and a reduced overall AF burden over 6 months of follow‐up (Voskoboinik et al., 2020). Another recently published study aimed to determine whether abstinence or reduced alcohol intake lowers the AF risk in heavy drinkers (>60 g/day for men and >40 g/day for women). The results indicated that complete abstinence can reduce AF incidence by 62% compared to sustained heavy drinkers, while merely reducing intake did not provide a significant benefit. The protective effect was most evident in individuals with normal BMI and without underlying conditions such as DM, hypertension, stroke, HF or coronary artery disease (Lee et al., 2024). Furthermore, in a prospective study using high‐density LA mapping, alcohol consumption was independently associated with higher AF recurrence after a single ablation despite no significant differences in LA voltage or conduction properties between drinkers and abstainers. These findings suggest that alcohol adversely affects post‐ablation outcomes through mechanisms beyond detectable atrial substrate remodelling (Sagawa et al., 2022).

These findings highlight alcohol reduction as an effective, modifiable strategy in AF management. Accordingly, clinical care should emphasize patient‐centred interventions, including structured counselling, behavioural support and routine assessment of alcohol intake as part of comprehensive AF management. Equally important is patient education regarding the benefits of alcohol cessation, the CV risks associated with continued alcohol consumption, and the potential benefits of reduction or abstinence in improving rhythm outcomes and reducing AF burden (Piano et al., 2025). In parallel, governments should adopt policies such as increasing alcohol taxation, implementing minimum pricing, and restricting the hours per day or days of the week for purchasing alcoholic beverages (Kilian et al., 2023).

### 4.4 Cardiorespiratory fitness improvement

Numerous prospective observational studies indicate that regular exercise of modest intensity can decrease the onset and recurrence of AF relative to inactivity (Mishima et al., 2021; Morseth et al., 2016). Current evidence has shown an inverse correlation between cardiorespiratory fitness (CRF) improvement and AF risk (Garnvik et al., 2019; Jae et al., 2019; Steell et al., 2019). However, this relationship appears to have an upper threshold, beyond which high‐intensity exercise is associated with less favourable AF outcomes (Myrstad & Elliott, 2025). Indeed, other studies, primarily involving non‐elite endurance athletes, indicate that long‐term, high‐volume training is associated with at least a twofold higher risk of AF compared to non‐athletes, in a dose‐dependent manner (Johansen et al., 2022; Karjalainen et al., 1998; Myrstad et al., 2014).

Evidence from large cohort indicates a U‐shaped relationship between CRF and AF risk. The lowest risk appears at moderate CRF levels, around 10–12 metabolic equivalents (METs), while very high fitness, above approximately 13–16 METs, is associated with an increased incidence of AF (Andersen et al., 2015; Jae et al., 2019; Kunutsor et al., 2024; Qureshi et al., 2015; Steell et al., 2019). These findings suggest that while regular exercise is generally protective, extremely high levels of CRF may attenuate cardioprotective benefits and potentially enhance AF risk. Proposed underlying mechanisms involve increased inflammation, heightened vagal tone, and adverse atrial remodelling (Cerqueira É et al., 2020; Scott & Li, 2024; W. Wang et al., 2024). Of note, short and medium‐term exercise regimens decrease AF burden and recurrence and alleviate symptoms in patients with known history of AF. Thus, exercise represents a fundamental aspect of secondary prevention efforts (Elliott et al., 2023).

Last, in a multicentre RCT, a structured intervention using activity trackers and motivational calls increased physical activity after pulmonary vein isolation (PVI) but did not reduce atrial arrhythmia recurrence compared with activity tracking alone. These findings suggest that while physical activity improves post‐ablation, additional behavioural support may not translate into further reductions in recurrence of AF (Seifert et al., 2024).

### 4.5 Integrated risk‐factor packages

The 2024 European Society of Cardiology (ESC) guidelines for the management of AF positions lifestyle and risk factor modification (LRFM) as a central component of comprehensive AF management aimed at improving clinical outcomes (Van Gelder et al., 2024). Although these contributors to AF development and progression are well recognized, their targeted role in reducing AF‐related morbidity has only recently gained broader recognition (Lip et al., 2023; Pathak et al., 2014, 2015; Rienstra et al., 2018; Teraoka et al., 2024).

Traditional healthcare models, which are typically organized around single organ systems or conditions, may not adequately manage the complexity arising from the numerous risk factors associated with AF. Indeed, 4 out of 10 healthcare professionals reported that their healthcare system is poorly structured to provide effective LRFM in patients with AF (Millis et al., 2025). To address these barriers, local governments should prioritize the development of integrated AF risk factor management clinics that break down traditional silos. These AF‐focused clinics should include a multidisciplinary team of specialists, such as cardiologists, endocrinologists, sleep physicians, exercise physiologists, dietitians, psychologists and other allied health providers. Such a coordinated approach facilitates holistic evaluation and management of AF, advancing care continuity, patient compliance and enhanced clinical outcomes (Hendriks et al., 2012).

## 5 PERI‐ABLATION STRATEGY: OPTIMIZING SUBSTRATE BEFORE AND AFTER PVI

### 5.1 Prehabilitation window

Catheter ablation is a primary strategy to restore and maintain sinus rhythm in symptomatic paroxysmal and persistent AF (Van Gelder et al., 2024), yet outcomes remain suboptimal, particularly in long‐standing AF (Brooks et al., 2010). Progressive atrial substrate remodelling, driven by cardiometabolic and sleep‐related comorbidities, promotes the development and persistence of AF (Abed et al., 2013; Dimitri et al., 2012; Lau et al., 2010). Addressing these drivers is therefore essential for durable rhythm control, although current guidelines provide limited guidance on the optimal timing of lifestyle interventions relative to PVI.

A growing body of research now supports the incorporation of lifestyle modification before catheter ablation. ARREST‐AF (Aggressive Risk Factor Reduction Study for Atrial Fibrillation) was the first robust study to investigate this strategy (Pathak et al., 2014). It was a cohort study involving 281 consecutive patients scheduled for AF ablation, all of whom had a BMI of at least 27 kg/m^2^ and at least one CV risk factor. These patients were offered a structured risk‐factor modification programme based on the guidelines available at the time the study was conducted. The targeted factors included excess weight, hypertension, dysglycaemia, dyslipidaemia, alcohol consumption and tobacco use. Participants of the intervention group achieved notable reductions in body weight, BP, HbA1c and lipid levels. When compared with a group that did not undergo any risk‐factor modification, they experienced significantly lower rates of AF recurrence, as well as reduced AF burden and severity in cases where AF recurred. Single‐procedure, arrhythmia‐free survival was 32.9% in the intervention group compared with 9.7% in the control cohort, while arrhythmia‐free survival after the final ablation was 87% versus 17.8%, respectively. In addition, the reduction in the LA volume index was significantly greater in the intervention group than in controls. Despite being a non‐randomized study and therefore susceptible to selection and observer bias, this study provided compelling evidence suggesting that comprehensive risk‐factor management may substantially improve outcomes.

Beyond the observational findings of ARREST‐AF, the first randomized data come from the POP‐AF trial, which evaluated the efficacy of a nurse‐led integrated lifestyle programme on AF ablation outcomes (Vermeer et al., 2025). In this prospective, controlled study, patients referred to their first catheter ablation were randomized either to standard pre‐ablation counselling by the treating electrophysiologist or an integrated lifestyle programme, which included sleep apnoea testing, weight reduction, alcohol moderation, smoking cessation, and optimization of hypertension and dyslipidaemia prior to PVI. Among 145 patients (70 control, 75 intervention), the primary composite endpoint, hospitalizations for repeated ablations and direct current cardioversions over 12 months, occurred in 52 events in the control group [incidence relative risk (RR) 0.49, 95% confidence interval (CI) 0.30–0.78, *P* = 0.004]. Both repeat ablations (RR 0.43, 95% CI 0.18–0.94, *P* = 0.045) and direct current cardioversions (RR 0.52, 95% CI 0.28–0.92, *P* = 0.031) were significantly reduced in the intervention arm. These results demonstrate that structured, nurse‐led lifestyle modification before AF catheter ablation can halve the risk of repeat procedures and cardioversions within 1 year.

**FIGURE 2:**
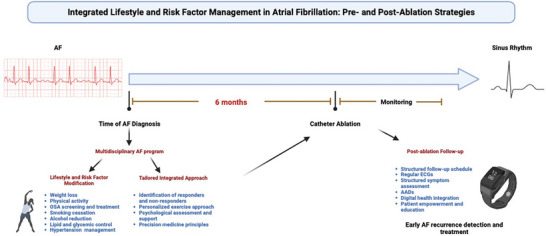
Proposed integrated management of patients with AF undergoing catheter ablation. Following AF diagnosis, patients should enter a multidisciplinary programme targeting lifestyle and risk factor modification (weight, physical activity, OSA, smoking, alcohol, lipids, glycaemic control, hypertension) and a tailored integrated approach (responder identification, personalized exercise, psychological support, precision medicine). At 6 months, catheter ablation may be performed even if the predefined targets have not been fully achieved, followed by structured post‐ablation follow‐up including ECG monitoring, symptom assessment, management of AADs, digital health integration and patient education. This strategy aims to optimize ablation outcomes and enable early detection of AF recurrence. AAD, antiarrhythmic drug; AF, atrial fibrillation; ECG, electrocardiogram; OSA, obstructive sleep apnoea.

Furthermore, findings from the POP‐AF trial illustrate that even a relatively short period of lifestyle optimization, approximately 5 months on average, can lead to meaningful improvements in ablation outcomes, despite many patients not achieving guideline‐based targets. In this study, weight reduction was the most prevalent intervention, and although only half of the patients with elevated BMI achieved ≥10% weight loss, the median reduction of just 5.5% was still associated with significantly lower rates of repeat ablation and cardioversion. These results challenge the assumption that only large weight reductions (≥10%) translate into clinical benefit and confirm that moderate changes are both feasible and effective in routine practice. Importantly, implementing lifestyle modification did not delay the ablation itself, supporting the practicality of pre‐procedural intervention. In contrast, previous trials such as SORT‐AF demonstrated that initiating risk‐factor management after ablation offers little benefit, suggesting that the window for modifying substrate and improving outcomes is before PVI (Gessler et al., 2021). Moreover, a recently published meta‐analysis indicated that performing catheter ablation within 1 year of diagnosis is linked to a lower risk of recurrence (Karakasis et al., 2025). This interval allows for the implementation of structured lifestyle interventions without compromising procedural timing and effectiveness. Taken together, current evidence supports integrating nurse‐led, multidisciplinary lifestyle programmes ahead of ablation rather than deferring intervention until after the procedure (Figure 2).

### 5.2 In‐procedure and post‐procedure implications

PVI represents the cornerstone of AF catheter ablation (Van Gelder et al., 2024). Because reconnection of previously isolated pulmonary veins is the most frequently identified mechanism of AF recurrence, ensuring durable PVI is essential for minimizing the risk of relapse (Ouyang et al., 2005; Verma et al., 2005). First‐pass isolation (FPI) during PVI typically provides a reliable metric of the quality of the initial radiofrequency encirclement and serves as a predictor of successful ablative results in paroxysmal and persistent AF (Luo et al., 2025; Osorio et al., 2021). Of note, the absence of FPI on the right pulmonary veins and posterior wall at index PVI was linked to 13‐fold and 10‐fold risk of chronic reconnection at repeat ablation, respectively (Pérez‐Pinzón et al., 2024).

Progressive atrial substrate remodelling in AF, often seen in cardiometabolic diseases, may compromise the likelihood of achieving FPI. Lower global LA voltage, indicative of fibrosis, has been associated with unsuccessful FPI (Garg et al., 2020). In patients with cardiometabolic disorders, catheter ablation strategies should therefore be tailored to address the greater degree of atrial remodelling and procedural complexity in this population. The substrate‐based strategy evaluated in the ERASE AF trial may help overcome the adverse impact of atrial fibrosis, as targeted ablation of low‐voltage areas identified by high‐density mapping significantly reduced AF recurrences compared with conventional PVI alone (Huo et al., 2022). More broadly, electroanatomical mapping has a critical role in guiding ablation therapy by enabling precise identification of arrhythmogenic substrates and confirmation of durable PVI (Rolf et al., 2014). Recently, pulsed field ablation (PFA) has emerged as a promising energy source that enables efficient PVI using ultra‐rapid electrical pulses to induce cell death through irreversible electroporation, while demonstrating preferential myocardial tissue ablation and a favourable safety profile compared with thermal ablation (Reddy et al., 2023; Turagam et al., 2023; Wittkampf et al., 2018). Although current evidence suggests that routine electroanatomical mapping may not be required for PVI using PFA, its integration may still hold value in selected patients with cardiometabolic disease and advanced atrial remodelling, where refined substrate characterization could further optimize lesion delivery and long‐term outcomes, warranting further investigation (Badertscher et al., 2024).

Consistent with the impact of cardiometabolic burden on ablation outcomes, FPI has been shown to exhibit a negative linear relationship with increasing BMI, with obesity also conferring a higher risk of post‐procedural atrial tachyarrhythmia (AT) recurrence (Okamatsu et al., 2024). In obese patients, conscious sedation may further complicate the creation of durable LA radiofrequency lesions because of airway obstruction and unstable respiration. General anaesthesia may mitigate these challenges and has been identified as a positive independent predictor of FPI (Okamatsu et al., 2024). Collectively, these findings support the importance of pre‐ablation optimization of patients’ cardiometabolic risk factors to improve the success of catheter ablation procedures.

Moreover, in a large cohort study by Saksena et al. using Optum's deidentified Market Clarity Data, 23,323 patients were followed after index AF ablation (mean age 63 years; 67% men), the majority of whom had at least one cardiometabolic comorbidity such as hypertension, coronary artery disease, DM, OSA or HF. AAD use remained common post‐ablation, with 46.9% of patients receiving AAD therapy after the initial procedure. Even among those who were AAD‐naïve at baseline, 27.6% initiated treatment during follow‐up. Reliance on AADs increased further with repeated procedures, reaching 62.8%, 69.9% and 68.6% among patients undergoing two, three or four or more ablations, respectively. Amiodarone was the most frequently prescribed agent (Saksena et al., 2025). This widespread post‐ablation strategy of administering AADs has been supported by a recent meta‐analysis which confirmed that antiarrhythmic therapy after ablation is associated with a reduction in AF recurrence (Y. Wang et al., 2024). However, the considerable side effects of these agents must be taken into account. In the ARREST‐AF trial, patients who underwent risk factor management were more likely to remain AF‐free without AAD after either a single or repeated procedure, with 16% requiring ongoing therapy compared with 42% in the control group at a mean follow‐up of 42 months (Pathak et al., 2015). In the POP‐AF study, a reduction in AAD use was reported in both treatment groups. In the intervention group, 27.1% of patients were receiving flecainide at baseline, but this proportion fell to 6.7% at both 3 and 12 months post‐ablation (Vermeer et al., 2025). Consequently, it is reasonable to consider discontinuation of AADs on an individual basis once adequate lifestyle modification has been achieved and technically successful AF catheter ablation has been performed (Figure 2).

### 5.3 Care pathways and team

The peri‐ablation period remains difficult to manage with traditional approaches and demands strong physician and patient adherence, the incorporation of technological advances, and close follow‐up to optimize clinical outcomes. Several AF‐specific care pathways have aimed to improve AF management, with mixed effects on clinical outcomes but consistently enhancing adherence to guideline‐directed therapy (Hendriks et al., 2012; Stewart et al., 2015; Wijtvliet et al., 2020).

The adoption of new technologies for continuous patient monitoring may further support these efforts. The TeleCheck‐AF programme, developed during the COVID‐19 pandemic, used app‐based heart rate and rhythm monitoring to facilitate teleconsultations, reducing face‐to‐face visits by 80% without affecting emergency department attendance (Gawałko et al., 2024; Pluymaekers et al., 2021). Similarly, the mAFA‐II trial demonstrated that high adherence (92%) to a mobile health intervention lowered the composite outcome of stroke, death and hospitalization (Guo et al., 2020). In contrast, the smaller emPOWERD‐AF study (*n* = 80) achieved only 53.9% app adherence but improved quality of life without altering AF‐related hospitalizations (Lazaridis et al., 2022). In another study, 64% of patients engaged with the AF‐specific mobile application monthly, leading to shorter times to arrhythmia recurrence and antiarrhythmic intervention over 2 years (Almeida et al., 2025). Early rhythm control is critical, and structured digital follow‐up may provide a scalable method to achieve this, though further research is needed to assess long‐term clinical benefits (Figure 2).

## 6 METHODOLOGICAL CONSIDERATIONS WHEN INTERPRETING LIFESTYLE EVIDENCE

As extensively discussed in this review, lifestyle modification is a cornerstone of AF management and is associated with improved ablation outcomes, supported by an expanding body of evidence from randomized trials and observational studies (Fitzgerald et al., 2022). However, interpreting this literature requires careful attention to methodological nuances. Confounding by indication is one of the primary challenges of cohort studies and occurs when a factor that influences the outcome is also a reason why certain individuals are selected for an intervention. As a result, differences in outcomes between exposed and non‐exposed groups may partly reflect underlying differences in baseline characteristics or clinical indications (Salas et al., 1999). Adherence bias is a similarly significant issue, with patients who are successful at following lifestyle interventions also being likely to adhere to other health promotion activities, confounding the intervention's effect itself. To address these barriers, researchers usually use advanced statistical techniques, such as propensity score matching, to create balanced groups (Kurz et al., 2024). Additionally, reverse causality may arise, for instance, if patients with milder AF or fewer comorbidities are more likely to participate in lifestyle programmes, artificially inflating apparent benefits. Reverse causality is often overlooked and should be considered as a potential explanation when apparent, often unexpected, interplay between risk factors and adverse outcomes occurs (Sattar & Preiss, 2017).

Intention‐to‐treat (ITT) analysis is considered the gold standard for RCTs and compares the treatment groups and involves all patients based on their original randomization, regardless of the actual treatment received. The per‐protocol analysis compares only those who fully completed the protocol, and while it estimates the maximum effect of the intervention, it often leads to bias. In noninferiority trials, both ITT and per‐protocol analyses, are recommended (Shah, 2011). Reliance on self‐reported lifestyle exposures, rather than objective measures, introduces further risk of misclassification. For instance, a large prospective population‐based cohort study of nearly 99,000 adults examined whether lifestyle behaviours predict new‐onset AF. Using standardized baseline questionnaires, they assessed self‐reported behaviours including physical activity, diet and sleep quality. While traditional risk factors such as age, sex, BMI, HF and prior stroke were associated with incident AF, baseline self‐reported lifestyle factors showed no significant relationship. The reliance on questionnaires at a single time point may partly explain these null findings (Siland et al., 2020). Objective monitoring using wearable devices, accelerometers or telemonitoring platforms can mitigate these biases by providing continuous and verifiable data on physical activity, weight change or sleep patterns (Gawałko et al., 2020).

Outcome assessment also presents notable challenges in AF studies. Endpoints range from time‐to‐first recurrence to cumulative AF burden, which are not interchangeable. For many years, the primary endpoint of most clinical studies conducted to investigate pharmacological and nonpharmacological treatments for AF has been the time‐to‐first recurrence of any atrial tachyarrhythmia, typically at 1 year of follow‐up. The selection of this endpoint is associated with certain limitations, which mainly arise from the assumption that all arrhythmia episodes carry equal clinical significance (Andrade et al., 2025). AF burden, defined as the percentage of time a patient is in AF, better reflects overall clinical status, including quality of life and hospital use. Its assessment, however, requires continuous monitoring, which is invasive and costly. Recent evidence shows that early recurrence of atrial tachyarrhythmia after rhythm control predicts higher long‐term AF burden, with most failures occurring within the first month, and that shorter follow‐up is feasible and cost‐saving (Andrade et al., 2025).

To overcome these issues, future research should incorporate innovative methodological approaches. Target‐trial emulation can strengthen causal inference in observational datasets by closely mimicking randomized trial conditions, ensuring comparability between intervention and control groups (Hubbard et al., 2024). Pragmatic RCTs embedded within routine clinical practice can capture real‐world adherence patterns, comorbidities and variations in healthcare delivery, increasing external validity, while providing cost advantages over traditional RCTs (Omerovic et al., 2024). Factorial designs allow the simultaneous evaluation of multiple lifestyle interventions, enabling assessment of independent and synergistic effects (Andrade, 2024).

Ultimately, the multifactorial nature of AF requires rigorous study design, including objective exposure assessment, standardized outcomes and robust trials, to clarify the impact of lifestyle interventions on prevention, ablation success and long‐term rhythm control.

## 7 IMPLEMENTATION, EQUITY, AND DIGITAL TOOLS

Cardiac rehabilitation (CR) improves long‐term cardiovascular outcomes by reducing mortality and hospitalizations and enhancing quality of life, through exercise, education, risk‐factor management and psychosocial support (Anderson et al., 2016; McGregor et al., 2020). Integrating CR principles into arrhythmia clinics, including prehabilitation before AF ablation, can further improve patient outcomes (Pathak et al., 2014; Vermeer et al., 2025).

However, in a recent survey of European healthcare professionals, responders reported that only 10% of patients with AF are directed to exercise‐based CR programmes. Key barriers include programme restrictions, limited patient motivation and unclear referral processes. Expanding AF‐specific exercise programmes and implementing strategies to enhance patient engagement, including education and digital tools, could improve participation and completion (Millis et al., 2025). Furthermore, the role of dietary interventions is often underestimated as an important component of holistic management for AF. The integration of nutritional counselling into everyday clinical practice may improve AF clinical outcomes (Nabil et al., 2024).

As a modifiable risk factor, the impact of poor sleep on AF is now acknowledged by physicians and leading organizations (Deshmukh et al., 2025). Screening of OSA is recommended in patients with AF, as its treatment improves clinical outcomes (Van Gelder et al., 2024). Beyond OSA, other types of sleep disorders, including insomnia, sleep deprivation, central sleep apnoea, and restless legs syndrome, have also been associated with the development of AF and its related complications (Deshmukh et al., 2025). The complex interplay between sleep and AF underscores the need to establish sleep management as a key component of CR, with involvement of sleep specialists.

Regarding OSA treatment, CPAP adherence is estimated to be nearly 50% (Pépin et al., 1999; Weaver et al., 1997). Education of the patient, different delivery machines and dedicated follow‐up can enhance compliance to CPAP (Pépin et al., 1999). However, this approach does not seem to apply in daily clinical practice. Among patients with AF and on CPAP therapy, only 23.8% of European healthcare professionals always emphasize CPAP adherence (Millis et al., 2025). Last, a major issue affecting patient access to CPAP therapy is its cost, highlighting the need for the healthcare system to take measures to protect economically vulnerable patients (Pandey et al., 2020).

AF imposes a significant cost burden on the healthcare system (Peigh et al., 2024). Previous findings indicate that an organized physician‐managed risk factor modification programme is effective clinically and cost‐efficient (Pathak et al., 2017). It is also reasonable to consider that a relatively low‐cost, multicomponent lifestyle programme that reduces the risk of a high‐cost re‐ablation procedure is likely to be a cost‐effective strategy. The data from the POP‐AF trial, which showed a reduction in the rate of repeat catheter ablation, seem to support this hypothesis by preventing costly reinterventions (Vermeer et al., 2025). Nevertheless, RCTs are warranted to evaluate the cost‐effectiveness of these treatment strategies.

The integration of wearable devices, including smartwatches, in daily clinical practice may provide physicians with useful information about patients’ AF burden, especially in the post‐ablation period. Indeed, these devices appear to be highly reliable in recording AF burden, demonstrating a low mean error compared to reference ECG monitoring methods, as shown in a recent meta‐analysis (Anagnostopoulos et al., 2025). Moreover, they improve the quality of information conveyed from patients to clinicians, which allows a more accurate assessment of overall clinical status, and should therefore be adopted as an additional valuable tool in AF‐specific clinics. However, ongoing efforts to reduce false‐positive alerts through artificial intelligence (AI) and improved algorithms remain essential, as false notifications have been shown to negatively impact health‐related quality of life and chronic disease self‐management (Tran et al., 2023). Research indicates that a structured digital‐blended follow‐up approach, mainly based on telemedicine, led to earlier identification of AF recurrence and quicker initiation of antiarrhythmic treatments compared to standard care. These findings underscore the potential benefits of incorporating digital tools for monitoring patients after AF ablation (Almeida et al., 2025).

## 8 FUTURE DIRECTIONS AND RESEARCH AGENDA

Lifestyle changes are no longer peripheral to AF management. Emerging evidence reinforces lifestyle modification as a key determinant of AF progression and an enhancer of rhythm control, including ablation, but mechanistic clarity, standardized outcomes and robust randomized data remain limited.

The absence of clearly defined, targetable lifestyle goals proven to improve ablation outcomes limits clinicians’ ability to structure effective multidisciplinary AF programmes. Consequently, further research is needed to establish evidence‐based endpoints for each modifiable risk factor and guide their implementation in clinical practice. Moreover, standard lifestyle interventions may not be sufficient for all patients to reach their optimal targets. Phenotype‐tailored lifestyle interventions for each patient, addressing the specific physiological and behavioural mechanisms underlying their condition rather than applying a uniform set of recommendations can improve adherence and effectiveness. Indeed, in patients with BMI ≥30 kg/m^2^, this approach produced significantly greater weight loss over a 3‐month period compared with a standard weight loss programme (Cifuentes et al., 2023).

It thus appears that individual responses to usual lifestyle interventions may vary, underscoring the limitation of the traditional ‘one‐size‐fits‐all’ approach. Precision medicine can address this issue by integrating genetic, physiological and behavioural factors to personalize treatment and optimize outcomes (Collins & Varmus, 2015; Gameiro et al., 2018). Emerging multi‐omics technologies, including genomics, proteomics, metabolomics, microbiome analysis and epigenomics, provide new avenues to improve our understanding of mechanistic pathways in patients with AF (Anazco & Acosta, 2025; Karakasis et al., 2025). By leveraging these tools, management of lifestyle risk factors for AF can evolve toward a more personalized, mechanism‐driven strategy aimed at reducing AF burden and preventing post‐ablation recurrence (Molla & Bitew, 2024). RCTs are warranted to validate this hypothesis and guide clinical practice.

Beyond ensuring that lifestyle interventions achieve their targets, it is also necessary to determine the optimal timing of their implementation in relation to catheter ablation. Ideally, interventions should be applied before the development of an irreversible arrhythmogenic substrate. Recent advances in imaging, electroanatomic mapping, biomarker profiling, ECG analysis, and multi‐omics approaches can provide a comprehensive evaluation of atrial cardiomyopathy, facilitating early detection and personalized treatment strategies (Karakasis et al., 2025). Based on current data, initiating lifestyle modification prior to catheter ablation appears most appropriate (Vermeer et al., 2025), although further research is needed to reinforce this approach.

In addition, further research is warranted to identify agents that act synergistically with lifestyle interventions to optimize therapy. Among these, GLP‐1 receptor agonists, likely due to their pleiotropic effects, appear to reduce the risk of AF recurrence after catheter ablation (Karakasis et al., 2024).

Wearable technologies are poised to become integral to improving ablation outcomes, but their role must be defined through robust research. Smartwatches and similar devices can support lifestyle interventions by objectively tracking sleep patterns, physical activity and adherence. In the post‐ablation phase, they also enable reliable monitoring of AF burden and recurrence. Well‐designed studies are needed to determine which devices offer sufficient accuracy, which parameters should be prioritized, and how these data can be incorporated into structured AF care pathways. Establishing such evidence will be essential for wearable technology to meaningfully enhance both lifestyle modification and procedural success. In addition, AI is also reshaping AF management by personalizing therapy and improving ablation strategies. AI‐guided electroanatomical mapping and real‐time procedural support may increase ablation success and reduce recurrence, though its clinical effectiveness remains to be established (Karakasis et al., 2025). Finally, LA digital twin models are emerging as a transformative approach to precision therapy in AF. These models integrate patient‐specific anatomical, electrophysiological and haemodynamic data, enabling individualized assessment of arrhythmogenic mechanisms, thromboembolic risk and treatment response. Digital twins incorporate MRI or CT imaging to model tissue properties and fibrosis distribution, allowing simulation of AF and virtual testing of ablation strategies prior to the procedure. During procedures, real‐time integration of intraoperative data, including electrograms, catheter positioning and wearable monitoring, allows dynamic adjustment of ablation plans, enhancing precision and efficacy (Xie et al., 2026). For patients with cardiometabolic disease, who often exhibit advanced atrial remodelling, this multimodal, continuously updated approach can optimize lesion delivery, reduce AF recurrence, and support personalized management based on the patient's evolving physiological and molecular profile. Despite challenges in data integration and model validation, ongoing advances suggest significant potential for personalized AF management (Karakasis et al., 2025).

## 9 CONCLUSION

Currently, catheter ablation is widely utilized for rhythm control in AF, yet recurrence rates remain suboptimal, particularly among patients with modifiable lifestyle‐related risk factors. The expected rising demand for ablative procedures underscores the need to optimize procedural outcomes and explore adjunctive strategies. Lifestyle interventions targeting obesity, sleep apnoea, physical inactivity and alcohol consumption have demonstrated the potential to modify the arrhythmogenic substrate and improve post‐ablation success. When applied systematically and at the right time, lifestyle modification can serve as a co‐equal pillar with ablation, further enhancing procedural success and mitigating AF burden. However, variability in patient response highlights the importance of phenotype‐tailored approaches and standardized lifestyle endpoints. Future research is warranted to establish the effects and generalizability of lifestyle modification for AF.

## AUTHOR CONTRIBUTIONS

Konstantinos Grigoriou: Investigation, Formal analysis, Visualization, Project administration, Writing ‐original draft, Writing – review & editing. Paschalis Karakasis: Conceptualization, Methodology, Investigation, Visualization, Project administration, Writing ‐original draft, Writing – review & editing. Panagiotis Theofilis: Writing ‐original draft, Writing – review & editing. Konstantinos Pamporis: Writing ‐original draft, Writing – review & editing. Panayotis K Vlachakis: Writing ‐original draft, Writing – review & editing. Anastasios Apostolos: Writing ‐original draft, Writing – review & editing. Nikolaos Ktenopoulos: Writing ‐original draft, Writing – review & editing. Barbara Fyntanidou: Writing ‐original draft, Writing – review & editing. Efstratios Karagiannidis: Writing ‐original draft, Writing – review & editing. Dimitrios Patoulias: Writing ‐original draft, Writing – review & editing. Antonios P Antoniadis: Writing ‐original draft, Writing – review & editing. Nikolaos Fragakis: Conceptualization, Methodology, Investigation, Writing – review & editing, Validation, Supervision. All authors have read and approved the final version of this manuscript and agree to be accountable for all aspects of the work in ensuring that questions related to the accuracy or integrity of any part of the work are appropriately investigated and resolved. All persons designated as authors qualify for authorship, and all those who qualify for authorship are listed.

## CONFLICT OF INTEREST

None declared.

## FUNDING INFORMATION

None.

## Data Availability

All data generated in this research are included within the article.
